# Repositioning and optimization of left ventricular lead position in nonresponders to cardiac resynchronization therapy is associated with improved ejection fraction, lower NT-proBNP values, and fewer heart failure symptoms

**DOI:** 10.1016/j.hroo.2022.06.010

**Published:** 2022-06-30

**Authors:** Rasmus Borgquist, David Mörtsell, Uzma Chaudhry, Johan Brandt, Maiwand Farouq, Lingwei Wang

**Affiliations:** ∗ Cardiology, Department of Clinical Sciences, Lund University, Lund, Sweden; † Arrhythmia Section, Skane University Hospital, Lund, Sweden

**Keywords:** Cardiac resynchronization therapy, Heart failure, Left ventricular lead position, Clinical outcome, Reverse remodeling

## Abstract

**Background:**

Observational data suggest that an anterior or apical left ventricular (LV) position in cardiac resynchronization therapy (CRT) is associated with worse outcome and higher likelihood of “nonresponse.” It is not known whether the benefits of optimizing LV lead position in a second procedure outweighs the procedural risks.

**Objective:**

To evaluate the clinical effects of LV lead repositioning.

**Methods:**

During the period 2015–2020, we identified all patients in whom the indication for the procedure was LV lead repositioning owing to “nonresponse” in combination with suboptimal LV lead position. All patients were followed with a structured visit 6 months post LV lead revision. Heart failure hospitalization and mortality data were gathered from the medical records and cross-checked with the population registry.

**Results:**

A total of 25 patients were identified who fulfilled the inclusion criteria. All procedures were successful in establishing LV lead pacing in a lateral mid or basal location. Median follow-up was 2.5 years [1.1–3.7]. There were improvements in NYHA class (mean -0.5 ± 0.5 class, *P* < .001), LV ejection fraction (+5 [interquartile range 2–11] absolute %, *P* = .01), QRS duration (-36 [-44 to -8], *P* < .001) and N-terminal pro–brain natriuretic peptide (NT-proBNP) (-615 [-2837 to +121] ng/L, *P* = .03). Clinical outcome was similar to a reference population with CRT (*P* = ns).

**Conclusion:**

In nonresponders to CRT with either an anterior or inferior LV lead position, it was feasible to perform LV lead repositioning in all cases, with a low complication rate. Changing the LV lead position was associated with improved LV ejection fraction, larger QRS reduction, and larger NT-proBNP reduction.

**Key Findings**

▪ Left ventricular lead repositioning in cardiac resynchronization therapy is feasible, with high success rate and low complication rate, using modern implant tools.
▪ Repositioning the left ventricular (LV) lead from LV septal or apical segments to lateral segments is associated with improvements in QRS duration, N-terminal pro–brain natriuretic peptide levels, and NYHA class symptoms.
▪ Although marked symptomatic improvement was achieved in most cases, it is unknown whether these improvements in surrogate measures transform into better long-term clinical outcome.

## Introduction

Cardiac resynchronization therapy (CRT) is well validated for the treatment of heart failure in presence of systolic dysfunction and widened QRS duration.^1^ Over the years, selection of patients has evolved based on accumulating evidence, and current guidelines allocate a class Ia recommendation for patients with left bundle branch block (LBBB) and QRS width >150 ms.^2^ For patients with non-LBBB morphology or QRS duration between 130 and 150 ms, a class IIa or IIb recommendation is given. The percentage of patients who experience clinical benefit and/or improved systolic function is higher in the LBBB group, but even in this group there remains a minority of patients who do not derive any measurable benefit from the CRT treatment. The reason for this can be multifactorial, and one modifiable factor may be suboptimal left ventricular (LV) electrode position.^3^ Several post hoc and observational analyses have indicated that an anterior or apical LV electrode position is associated with worse clinical outcome and less improvement of LV function.^4^, ^5^, ^6^ In studies with tailored LV lead placement (late mechanical or electrical activation), the optimal site is most commonly situated in the basal or mid inferolateral or anterolateral region of the left ventricle.^7^^,^^8^ However, it is not known whether the benefits of optimizing LV lead position for nonresponders in a second procedure outweighs the procedural risks, for patients with apical and/or anterior lead position after the first implant. We sought to investigate this in a cohort of patients that, owing to nonresponse to CRT after the primary implant procedure, had undergone repositioning of the LV lead at our institution.

## Methods

From a larger cohort of 670 CRT-implanted patients at a tertiary referral center during the years 2015–2020, we identified all patients in whom the indication for the procedure was LV lead repositioning owing to “nonresponse” and suboptimal LV lead position. The initial CRT implant had been performed either at the same institution or at other referral institutions, and owing to either implanter inexperience or technical challenges, the LV lead had been implanted suboptimally, either anteriorly, inferiorly, or at the apex. During the initial implant, either a suitable vein was not visualized during the procedure (owing to lack of occlusive venogram or an overlooked early branch with retrograde filling), or the technique and tools selected did not permit implant in the most suitable vein. Five parameters were evaluated following each procedure: LV ejection fraction (EF) improvement ≥5% (absolute), NYHA class improvement (≥1 class), QRS duration reduction (≥20 ms), subjective physical improvement (“definitely yes” vs. “no definite improvement”), and reduction in N-terminal pro–brain natriuretic peptide (NT-proBNP) level (≥25% relative reduction). Patients with no subjective improvement and unchanged or worsened outcome on at least 3 of the other 4 criteria were defined as nonresponders and were offered a second procedure, if the LV electrode was in a nonlateral or apical position. Differential programming of the device settings by varying the AV and VV settings had been tried but had failed to result in any significant improvement. All re-do procedures were performed by experienced implanters (having performed more than 200 independent implants). Interventional CRT implant techniques, including venoplasty and snaring, were used at the operator’s discretion. Patients were seen at follow-up with a structured visit 6 months post LV lead revision. For comparison between the procedures, an overall response score was created by adding 1 possible point for each of the 5 response criteria above, to a maximum of 5 points. Clinical endpoint data were gathered from the medical records and cross-checked with the Swedish population registry. Follow-up included echocardiography (LV dimensions and EF were evaluated by Simpson’s biplane method), electrocardiogram, NT-proBNP level, and assessment of heart failure symptoms by NYHA class and subjective perceived improvement in physical capacity.

### Procedural details

A minimal incision was made at the cephalad part of the previous skin incision. Vascular access was gained through direct puncture of the axillary vein or subclavian vein, if needed guided by ultrasound or contrast dye injection. In case of venous occlusion, a venoplasty was performed or access was gained by first extracting an existing lead and using the same access for the new lead. Coronary sinus access was established with either a 9F Medtronic Attain Command Surevalve deflectable catheter (Medtronic, Minneapolis, MN) in combination with a bipolar CS-assist steerable electrophysiology catheter (Boston Scientific, Marlborough, MA), or with an 11F Worley Standard or Jumbo sheath (Merit Medical, South Jordan, UT). Sub-select catheters were used as needed, either Medtronic Select II Surevalve or Worley LVI Renal 5.5F in combination with a Worley vein selector (standard, hook or vertebral) from Merit Medical. A venogram was performed and an inferolateral vein was targeted as the first choice, and an anterolateral vein as the second choice, depending on availability. Quadripolar LV leads were used in all cases, threshold was tested, and phrenic nerve capture was avoided. If possible, without use of dedicated extraction tools, the old LV lead was then removed prior to slitting the sheaths and fixating the new LV lead. TYRX antibacterial envelope was used for all patients with high risk according to the PADIT risk score, or at the discretion of the implanter.^9^ Postoperatively AV and VV delays were optimized by testing of multiple settings to produce as narrow QRS duration as possible. CRT devices from Abbott (Sylmar, CA) or Medtronic were used. If the device had an LV-only pacing algorithm and the patient had sinus rhythm with normal AV conduction, the algorithm was tested, and QRS duration was compared to the QRS duration with biventricular pacing. The study was approved by the Swedish Ethical Review Authority. The data collection was part of a large patient cohort where anonymized data were collected from all CRT-treated patients at our institution, and the requirement for written informed consent was waived by the review authority (No. 2020-05843).

### Statistical methods

SPSS version 27 (IBM, Chicago, IL) was used for statistical analyses. Normally distributed data are presented as mean ± standard deviation; non-normally distributed data are presented as median [interquartile range]. McNemar’s test was used for paired comparisons between the first procedure and the LV repositioning procedure, for the prespecified categorical criteria such as LVEF response, and for the overall response score. Kaplan-Meier analysis with log-rank test was used to compare survival between the intervention cohort and a reference cohort. For all analyses, a 2-sided *P* value < .05 was considered significant.

## Results

**Figure 1:**
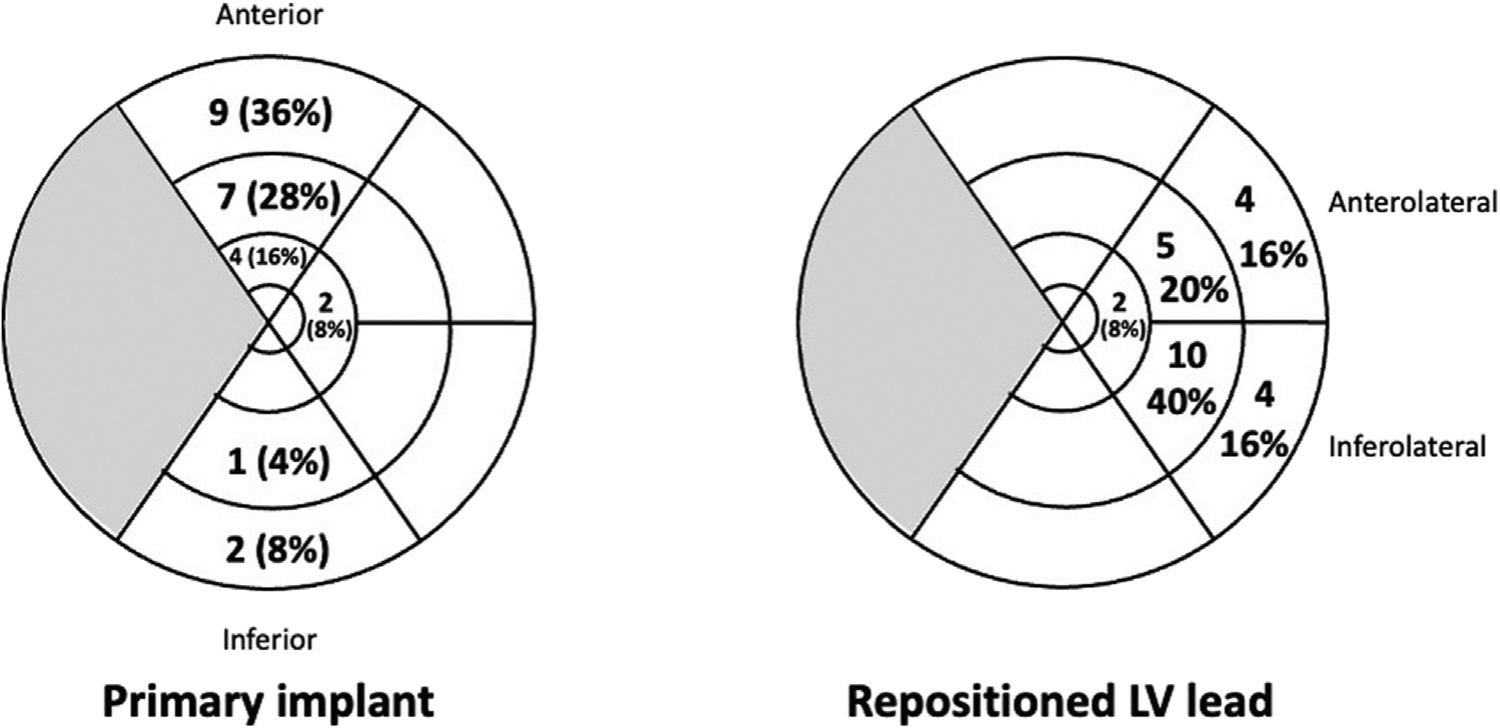
Left ventricular (LV) lead position in the 17-segment bulls-eye model. The left panel shows lead position after the first procedure; the right panel shows lead position after the second procedure. The outermost segments are basal, followed by mid segments, apical segments, and apex (the innermost circle). Gray segments are septal and white segments are anterior, anterolateral, inferolateral, and inferior in clockwise order from 12 o’clock in the circle.

**Figure 2:**
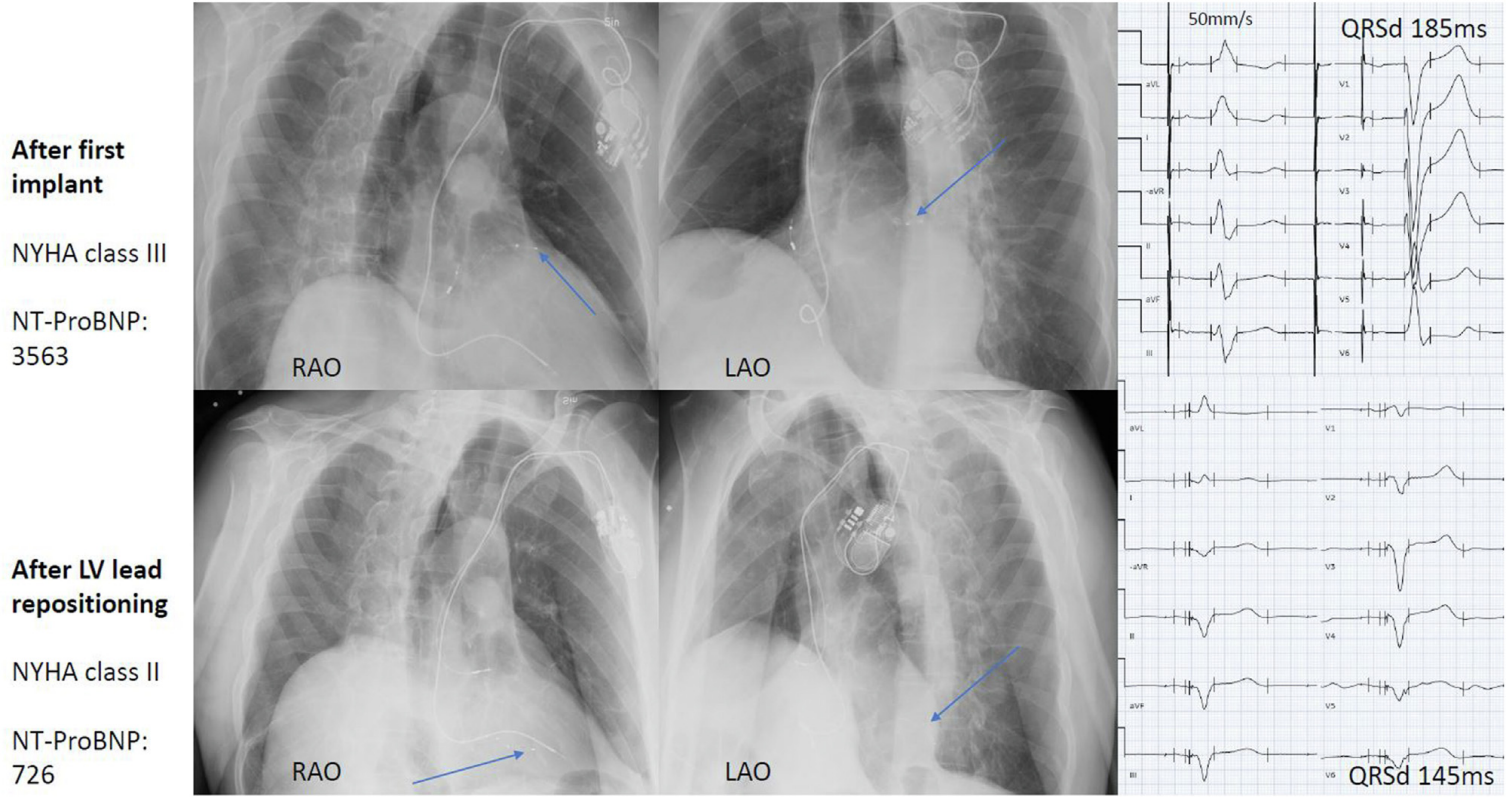
An 80-year-old male patient with ischemic cardiomyopathy and cardiac resynchronization therapy-pacemaker. Initial left ventricular (LV) lead position was basal anterior, and after repositioning the position was mid inferolateral. Changes in lead position, electrocardiogram appearance, NT-proBNP, and New York Heart Association (NYHA) class are illustrated in the figure. LAO = left anterior oblique; RAO = right anterior oblique.

In all, 25 patients were identified who fulfilled the inclusion criteria. Baseline characteristics at the time of the primary implant are presented in Table 1. The median time between the primary implant and the LV lead repositioning was 1.6 [0.9–4.5] years and after the revision procedure they were followed for a median of 2.5 [1.1–3.7] years. Median procedure time and fluoroscopy time for the primary implant was 81 [IQR 62–115] minutes and 18 [10–41] minutes, respectively. LV lead location and LV paced segment was primarily anterior after the primary procedure, and primarily inferolateral after the second procedure (Figure 1). All LV lead revision procedures were successful in establishing LV lead pacing at another location than that from the primary procedure. A representative case including x-ray images of the lead positions is shown in Figure 2.

**Table 1:** Baseline demography of patients (N = 25) at the time of first cardiac resynchronization therapy implant

	At first implant	At LV repositioning procedure
Age (years)	73 [66–77]	76 [67–80]
Female	2 (8%)	2 (8%)
CRT-defibrillator	20 (80%)	20 (80%)
Ischemic cardiomyopathy	10 (40%)	10 (40%)
Hypertension	14 (56%)	14 (56%)
Diabetes	7 (28%)	7 (28%)
Atrial fibrillation	9 (36%)	9 (36%)
ECG morphology		
LBBB	17 (68%)	
IVCD	5 (20%)	
Paced	3 (12%)	25 (100%)
NYHA class		
II	12 (48%)	4 (16%)
III	13 (52%)	21 (84%)
ACE inhibitor/ARB/ARNi	22 (88%)	25 (100%)
Beta blocker	23 (92%)	25 (100%)
Aldosterone antagonist	13 (52%)	15 (60%)
Digoxin	1 (4%)	1 (4%)
Loop diuretics	17 (68%)	17 (68%)
Anticoagulant therapy	12 (48%)	14 (56%)
LVEF (%)	28 [22–31]	25 [22–30]
QRS duration (ms)	168 [154–182]	180 [168–196]
NT-proBNP (ng/L)	1104 [612–2007]	2788 [856–3928]
S-creatinine (μmol/L)	94 [88–125]	121 [89–144]

Results are n (%) or mean [interquartile range].

ACE = angiotensin-converting enzyme; ARB = angiotensin receptor blocker; ARNi = angiotensin receptor neprilysin inhibitor; CRT = cardiac resynchronization therapy; ECG = electrocardiogram; IVCD = intraventricular conduction delay; LBBB = left bundle branch block; LV = left ventricular; LVEF = left ventricular ejection fraction; NYHA = New York Heart Association.

Programming was similar after both procedures; vendor-specific algorithms for AV and VV optimization were used. Four patients had atrial fibrillation; for the remainder the median AV time was 120 [IQR 100–130] ms vs 120 [105–140] ms, *P* = .62. The median LV preactivation was 15 [0–30] ms vs 0 [0–30] ms, *P* = .80. LV-only pacing was used in 2 patients after the first procedure and in 5 patients after the second procedure.

Median procedure and fluoroscopy time for the LV lead reposition procedure was 78 [51–108] minutes and 12 [4–17] minutes, respectively. There were no early complications to the LV lead reposition procedures, but there was 1 late local infection (n = 1 [4%]), which resulted in extraction of the system and replacement with a right-sided CRT implant (with the LV lead in the same position).

### CRT effect evaluation

**Figure 3:**
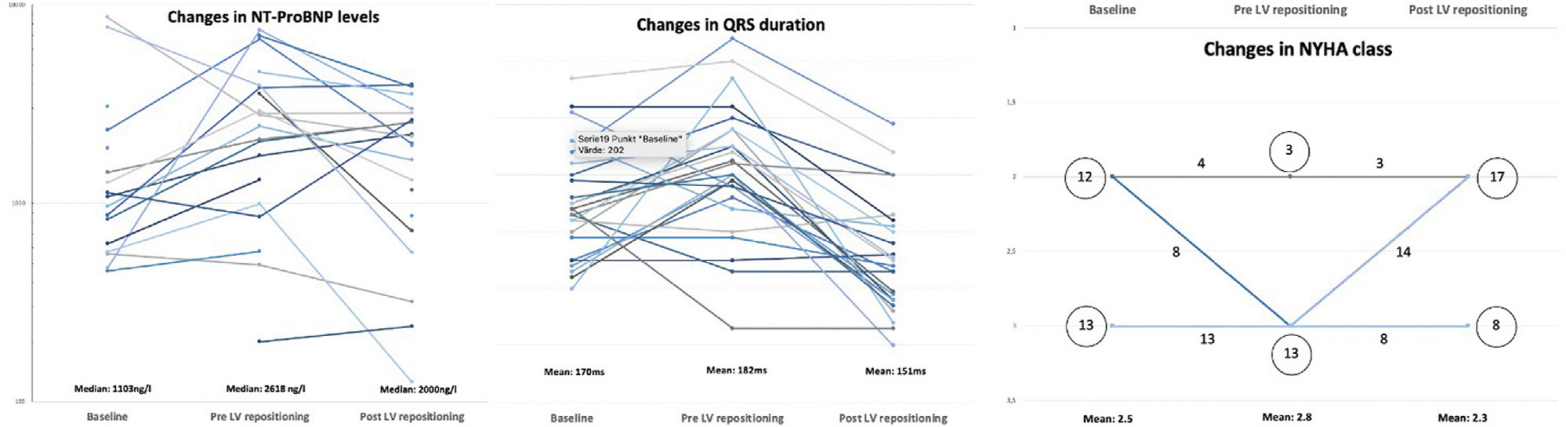
Changes in NT-proBNP levels, QRS duration, and New York Heart Association (NYHA) class between baseline, between procedure 1 and procedure 2, and after the left ventricular (LV) repositioning.

Overall, there were significant positive effects of the lead revision procedures; a summary of this is shown in Table 2. In Figure 3 the sequential changes in NT-proBNP levels, QRS duration, and NYHA class symptoms are presented. The proportion of patients who had a significant positive effect according to the 5 evaluated criteria was higher in all categories except NT-proBNP after the LV repositioning procedure; 71% vs 29% improved LVEF (*P* = .016), 72% vs 28% improved QRS duration (*P* = .013), 64% vs 18% improved NT-proBNP (*P* = .13), 52% vs 0% improved NYHA class (*P* < .001), and 48% vs 0% had a definite subjective improvement of physical capacity (*P* < .001). The mean overall response score was 2.7 ± 1.5 vs 0.7 ± 0.8 (*P* < .001). Central illustration presents a representative case showing significant improvements on both EF, NT-proBNP levels, and QRS duration reduction.

**Table 2:** Symptoms and diagnostic findings after the primary cardiac resynchronization therapy procedure, compared to after repositioning of the left ventricular lead

	Pre implant	After first implant	Change	Pre LV repositioning	After LV repositioning	Change	*P* value (for difference in delta)
NYHA class (average)			+0.2 ± 0.4			-0.5 ± 0.5	<.001
Class II	12 (48%)	8 (32%)		4 (16%)	17 (68%)		
Class III	13 (52%)	17 (68%)		21 (84%)	8 (32%)		
Patient-assessed response							<.001
Deterioration		3 (12%)			0		
No change		16 (64%)			8 (32%)		
Some positive effect		6 (24%)			4 (16%)		
Marked positive effect		0			12 (48%)		
Not evaluated		0			1 (4%)		
LVEF (%)	28 [22–31]	28 [22–30]	0 [-2 to +7]	25 [22–30]	30 [25–35]	+5 [+2 to +11]	.01
QRS duration (ms)	168 [154–182]	176 [151–190]	+2 [-23 to +22]	180 [170–196]	148 [136–163]	-36 [-44 to -8]	<.001
NT-proBNP (ng/L)	1104 [612–2007]	2192 [986–2708]	+359 [-147 to +1628]	2448 [924–3884]	1977 [686–2688]	-615 [-2837 to +121]	.03
Biventricular pace (%)		97.5 [87–99]			97.5 [88–99]		.9

Results are based on N = 25 patients; data are presented as n (%) or mean [interquartile range].

LV = left ventricular; LVEF = left ventricular ejection fraction; NYHA = New York Heart Association.

**Figure 4:**
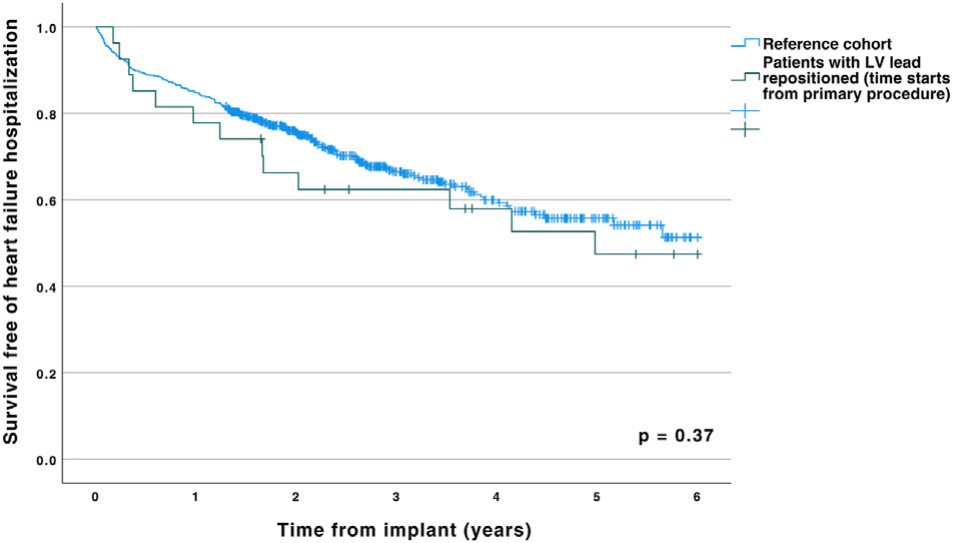
Kaplan-Meier curve of survival free of heart failure hospitalization, showing a reference cohort (blue, 550 consecutive cardiac resynchronization therapy cases) compared to the cohort with a primary procedure with suboptimal left ventricular (LV) lead placement and subsequent LV lead repositioning (*green*).

During the period between the first implant and the LV lead repositioning, 4 of the patients were hospitalized for heart failure, on a total of 8 different occasions. During follow-up after the LV lead repositioning, 3 patients were hospitalized on a total of 6 different occasions, and 4 patients died: 1 from heart failure, 1 from Covid-19 infection, 1 from renal and multiorgan failure, and 1 from sepsis not related to the device. Since there was no formal control group, a reference material consisting of 550 consecutive CRT-treated patients at the same institution during the period 2015–2020 was used to compare the clinical outcome of the present cohort with the “expected” outcome. The results are presented in Kaplan-Meier analysis in Figure 4. In a capped analysis at 2 years, the patients with nonoptimal LV lead position (mostly prior to revision, which occurred after a median of 1.6 years) had higher risk of heart failure hospitalization (log-rank test *P* = .03), but this difference then disappeared during longer follow-up, ie, presumably after the beneficial LV repositioning effect.

Over the entire follow-up period, measuring from the primary implant, the overall survival in the LV lead repositioning cohort was significantly better compared to the reference cohort in Kaplan-Meier analysis (log-rank *P* = .01), but in a landmark analysis starting from the second implant, this difference was not significant (*P* = .43).

## Discussion

In this case series of 25 patients we show that repositioning of the LV lead from an anterior or apical position is feasible and can be done with low complication rate. Furthermore, there were significant improvements on several measures traditionally associated with beneficial CRT effect, such as QRS reduction, NT-proBNP reduction, and NYHA class improvement. Whether or not this translates into longer-term clinical benefit with regard to mortality and risk of heart failure hospitalization cannot be determined owing to the limited sample size and the retrospective design of the study.

### The importance of LV lead location for successful CRT

The objective of CRT is primarily to restore electrical synchrony of the left ventricle, with the assumption that this transforms into better mechanical synchrony, more effective contraction, and, over time, reverse remodeling with reduced end-systolic volume and improved EF. Studies on electrical activation in patients with heart failure and LBBB have shown that the posterolateral basal and mid region is usually the latest activated part of the left ventricle. However, the electrically latest activated segment can vary, even in patients with classic LBBB, but more so in patients with atypical LBBB or nonspecific intraventricular conduction delay.^10^^,^^11^ Identification of the segment with latest mechanical activation has been evaluated by speckle-tracking echocardiography in several prospective trials. In the Imaging CRT trial, the optimal site was found in an anterior location in only 2 of 189 patients (1%) and inferior location in 1 patient (0.5%).^12^ In a similar study the percentage of anterior and inferior optimal locations were 7% and 8%, respectively.^7^ Early invasive studies showed that hemodynamic response varied significantly with LV lead position, and that a lateral position resulted in significantly better acute hemodynamic performance, compared to an anterior position.^13^ Acute hemodynamic improvement was later linked to positive long-term remodeling effect.^14^ Indeed, results from the MADIT-CRT study showed that clinical outcome (risk of death or heart failure hospitalization) was worse for patients where the LV lead was in an apical position.^5^ Similarly, in a large cohort of more than 2000 patients, it was shown that an anterior LV lead position was associated with unfavorable outcome.^6^

The heterogeneity with regard to both electrical and mechanical activation patterns in CRT-eligible patients with wide QRS implies that some patients can actually have an optimal LV lead position in the anterior or apical segments and derive significant benefit from CRT with the LV lead in this position. However, for the patients in our cohort, the initial LV lead position had not resulted in any improvement post-CRT. Other potential factors for nonresponse (device programming, scar tissue, anemia, medical therapy) had been excluded or deemed to be of less importance for the individual patient. Furthermore, the QRS appearance post-CRT had been evaluated and was either unchanged in morphology (ie, no visually altered activation pattern compared to intrinsic pattern) or significantly prolonged. In essence, there was a high *a priori* likelihood that repositioning of the lead could have a positive impact, and other influencing factors had been reasonably excluded. In such a cohort, we show that it is possible to obtain a significant improvement on several measures by repositioning the LV lead.

### Is improvement in surrogate measures good enough?

Reduction in QRS duration implies resynchronization of the ventricular activation, and larger reduction has consistently been associated with better outcomes,^15^, ^16^, ^17^, ^18^ but only 1 prospective randomized trial has used QRS narrowing as the target for optimization.^19^ In this trial the rate of echocardiographic remodeling was 74% in the QRS-optimized group vs 53% for the control group. Reductions in NT-proBNP or BNP have been associated with both short-term response and favorable longer-term clinical outcome.^20^^,^^21^ Improvement in subjective functional capacity and in NYHA classification are subject to reporting bias both from the patient and from the physician. Nevertheless, these parameters have been incorporated in many studies regarding prediction of CRT response and have both been associated with better clinical outcome.^22^ Overall, there is good evidence to support that if CRT results in positive effects on the above parameters, these changes are likely to transform into improved clinical outcome for the patient.

### Clinical implications

The clinical effect of CRT is highly dependent on patient selection. The *a priori* expected effect differs greatly between, for instance, a patient with high scar burden, EF <20%, atrial fibrillation, and nonspecific conduction delay, compared to a patient with dilated cardiomyopathy in sinus rhythm with a typical LBBB pattern. Our cohort was mixed, with 40% ischemic cardiomyopathy, 32% non-LBBB patients, and 36% with atrial fibrillation, and the beneficial effects were seen across all subgroups. The expected effect of CRT should always be evaluated on an individual basis, taking all the patient-specific factors into account, and a structured evaluation and correction of modifiable factors should be performed.^23^ This includes the use of device-integrated algorithms for AV and VV optimization. If, after this, there is a less-than-expected clinical effect of the CRT, and the patient has an LV lead position that is either apical or very close to the septum (anteriorly or inferiorly), then a repositioning should be attempted. The procedure should be performed by an experienced implanting physician, and care should be taken to minimize the risk of infection, including the use of local antibacterial envelope as appropriate.

### Limitations

This was a single-center retrospective study, with all the inherent limitations of such a design. Evaluation of survival was subject to selection bias, since the patients survived until the second procedure, even though they did not have any measurable benefit from the first CRT implant. All procedures were performed by high-volume experienced operators familiar with interventional CRT techniques, in a tertiary care setting at a large university hospital, and the results may not be generalizable to all implanting centers.

## Conclusion

In nonresponders to CRT with either an anterior, apical, or inferior LV lead position, it was feasible to perform LV lead repositioning in all cases, with a low complication rate. Changing LV lead position from apical//anterior/inferior to lateral was associated with improved LVEF, larger QRS reduction, and larger NT-proBNP reduction. Over time this may transform to better clinical outcome with regard to survival and heart failure hospitalizations, but larger prospective studies are needed to confirm these data.
